# Supplementary material for: Selective replication and vertical transmission of Ebola virus in experimentally infected Angolan free-tailed bats
Source: Nat Commun. 2024 Jan 31;15:925. doi: 10.1038/s41467-024-45231-0 (PMC10830451; doi:10.1038/s41467-024-45231-0)
Supplement: Supplementary file 1 — Supplementary Info [file 41467_2024_45231_MOESM1_ESM.pdf]

Supplementary Information

Selective replication and vertical transmission of Ebola virus in experimentally infected Angolan free-tailed bats

S.A. Riesle-Sbarbaro, G. Wibbelt, A. Düx, V. Kouakou, M. Bokelmann, K. Hansen-Kant, N. Kirchoff, M. Laue, N. Kromarek, A. Lander, U. Vogel, A. Wahlbrink, D. M. Wozniak, D.P. Scott, J. B. Prescott, L. Schaade, E. Couacy-Hymann, A. Kurth*.

*Corresponding author. Email: kurtha@rki.de

This PDF includes:

Supplementary Figures 1 to 11
Supplementary Tables 1 to 7
Supplementary Text

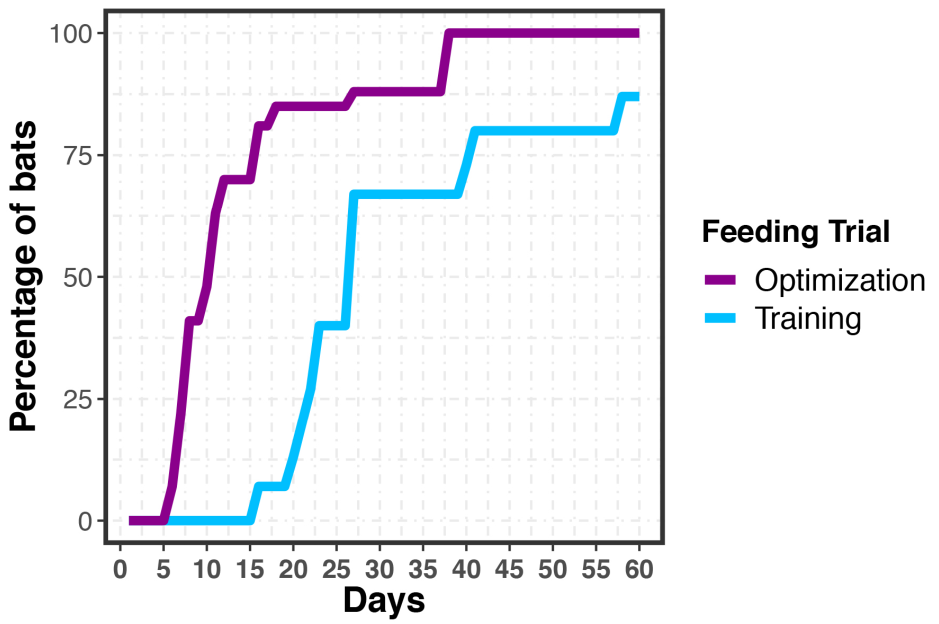

Supplementary Figure 1

Optimization of bat adaptation to a mealworm diet. Percentage of bats included in the pilot training (n=15 biologically independent animals) and the later optimization (n=27 biologically independent animals), which were able to self-feed from the dishes hanging at the flight-cage grid (Class 6, see also Table S1 and S2).

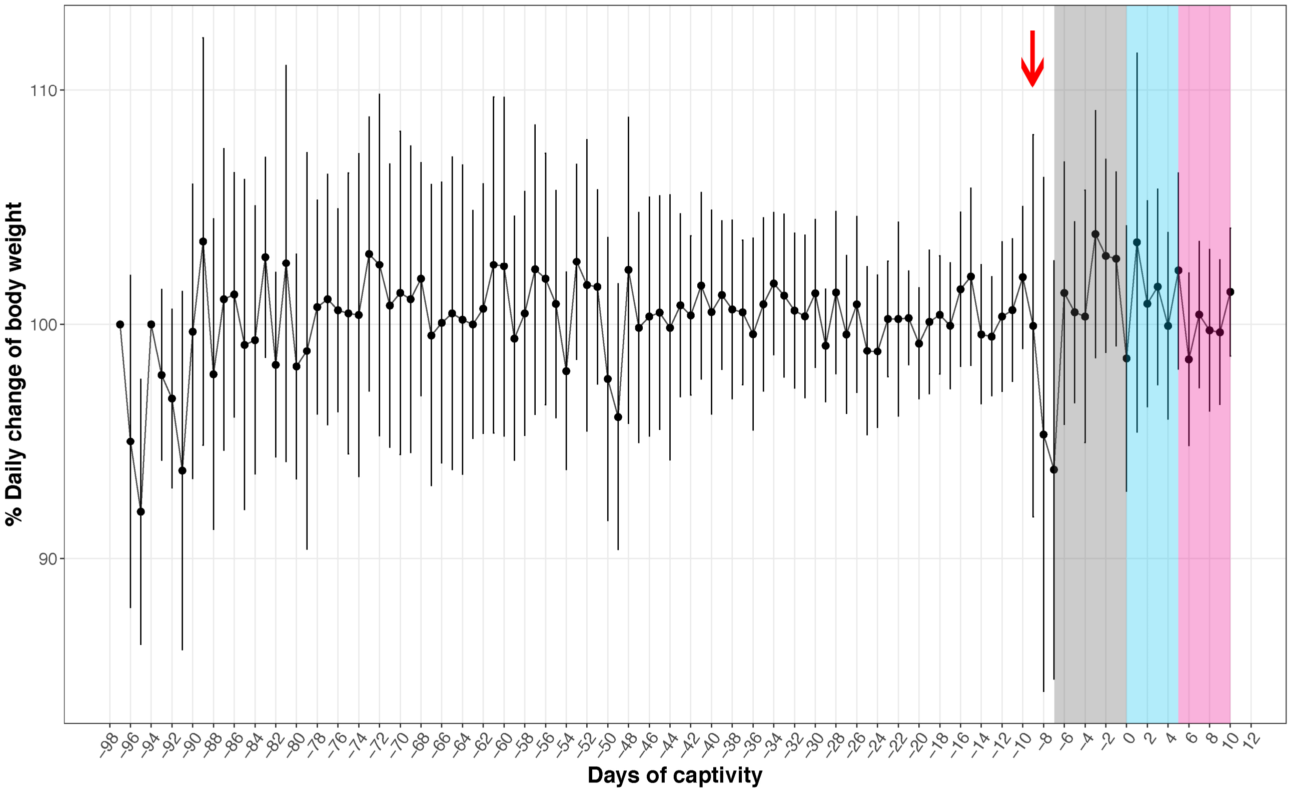

Supplementary Figure 2

Daily weight percentage change of all bats. Bats included in the study were measured from the captivity (no shade, n=37 biologically independent animals), acclimation (grey shade, n=33 biologically independent animals) and infection periods (blue, n=14 biologically independent animals and pink shades, n=12 biologically independent animals). Each point shows the mean value of the weight percentage change of that day ± one standard deviation (vertical line). The red arrow represents the day of air travel, with the following point of weight drop. The grey area denotes the beginning of the acclimation period, the blue area represents inoculation (0 dpi) to 5 dpi, and the pink area shows following days, until experimental endpoint: 10 dpi. No statistically significant differences were detected.

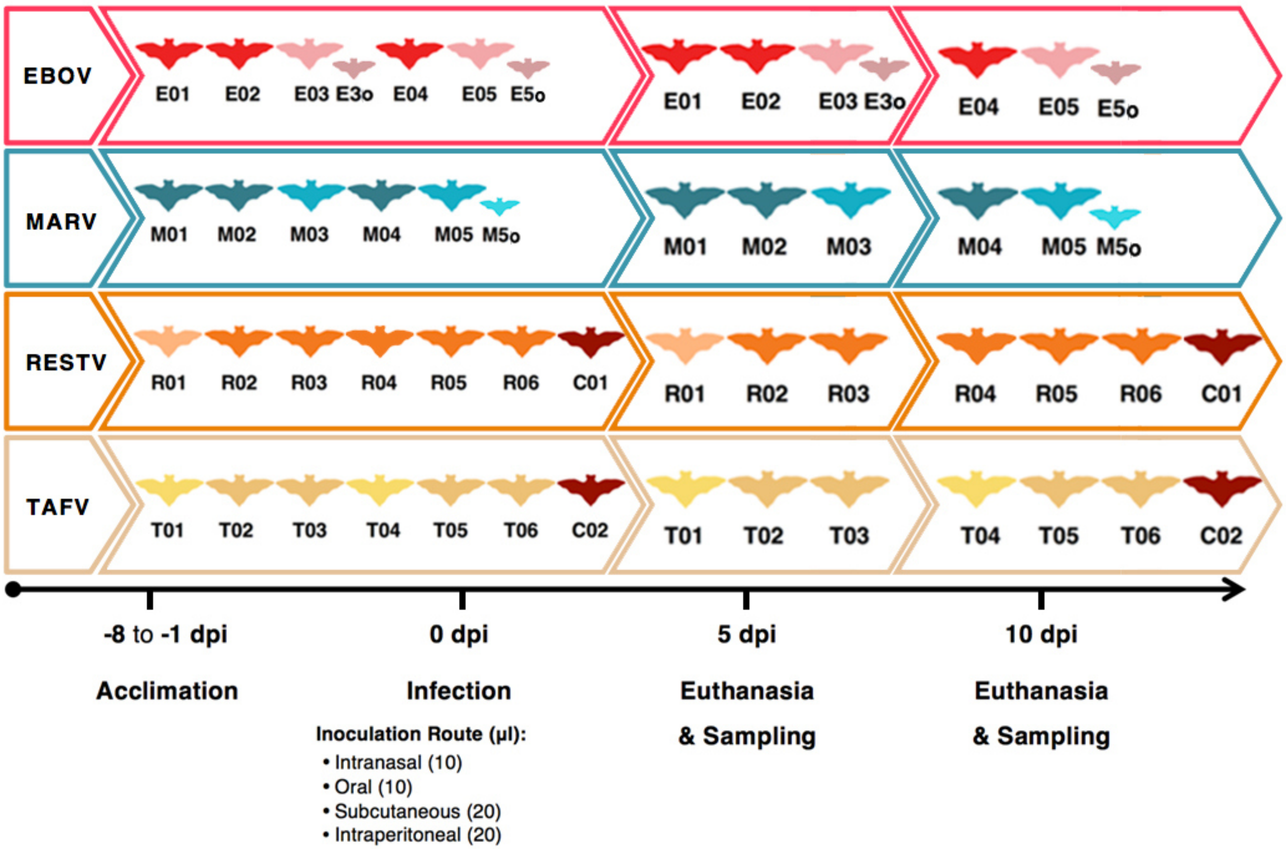

Supplementary Figure 3

Experimental design. Four filovirus cohorts are shown according to the experimental timeline; from the acclimation period to the infection endpoint. Colour coding show in red: EBOV, blue: MARV, orange: RESTV and beige: TAFV. Female bats are depicted in a lighter colour tone. Control bats are shown in brown.

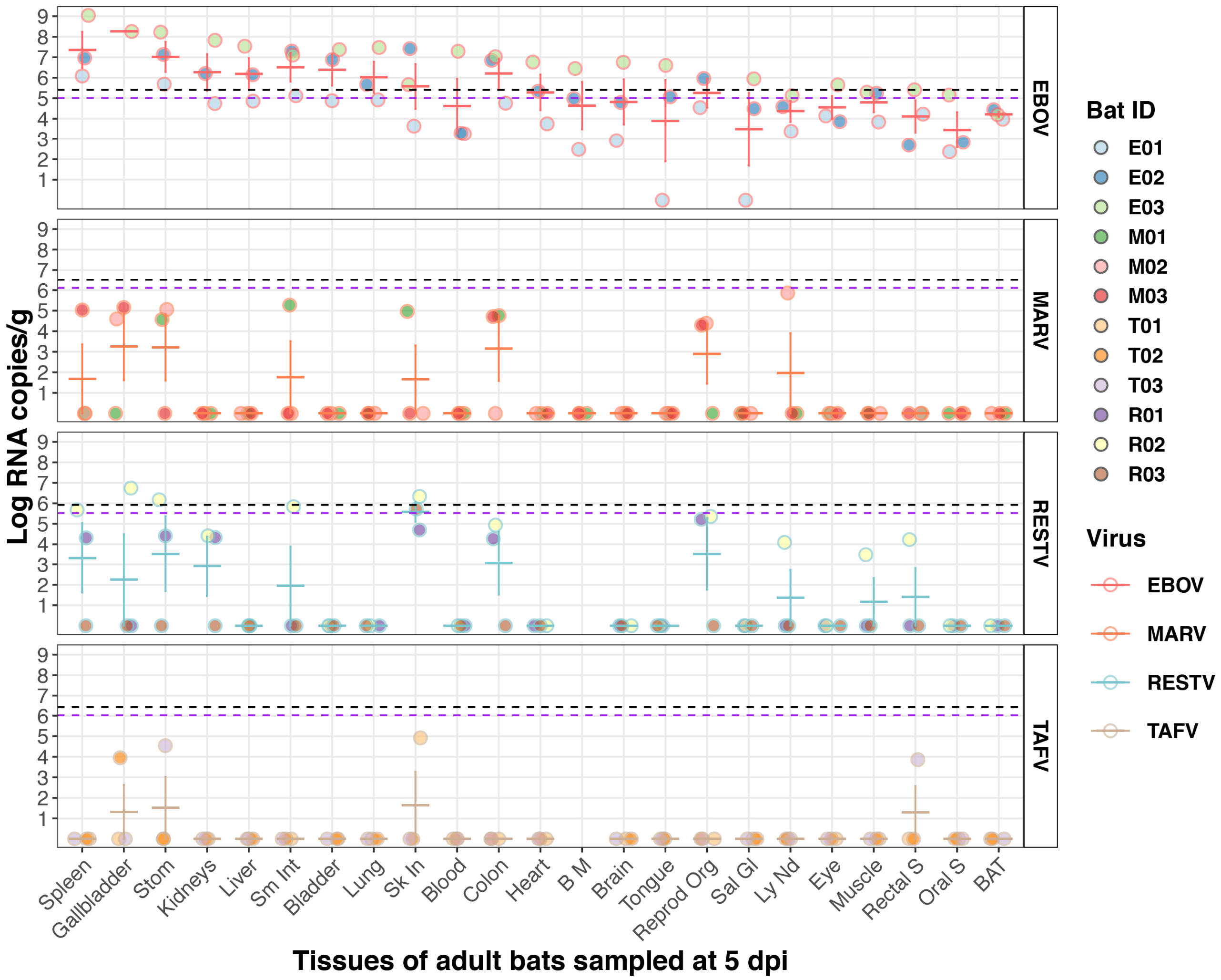

Supplementary Figure 4

Filoviruses RNA copies/gram of tissues from Angolan free-tailed bats (AFBs) sampled at 5 days post-inoculation (dpi). Distribution of each inoculated filovirus, shown as log-transformed RNA copies/gram of tissue or copies/ml of blood. Each coloured point represents one of the three bats sampled at the 5-dpi timepoint. Geometric mean of virus RNA copies/gram of tissue ± SEM are represented with horizontal and vertical error bars. Black dashed line represents the inoculum dose and in purple the effective-inoculum-dose (diluted by total blood volume), individual for each virus group. Only the gallbladder of EBOV-inoculated bat E03 was sampled at 5 dpi.

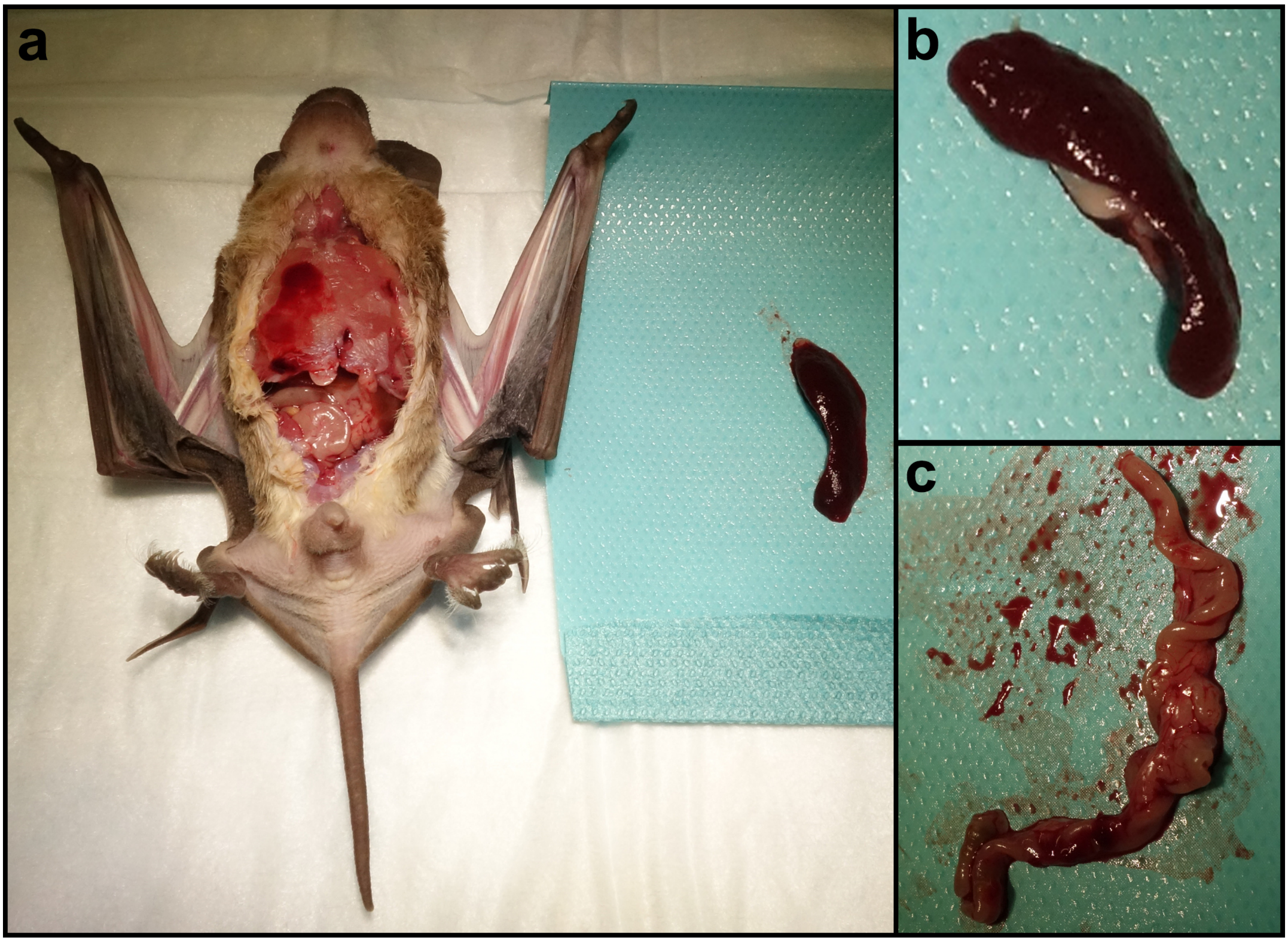

Supplementary Figure 5

Necropsy findings. a, Splenomegaly of the MARV-inoculated bat M01 and **b,** irregular margins of the spleens of bat M04. **c,** Large intestine with minor haemorrhage. Pictures are representative of one animal from 5 within the experimental group (dpi 5 and 10).

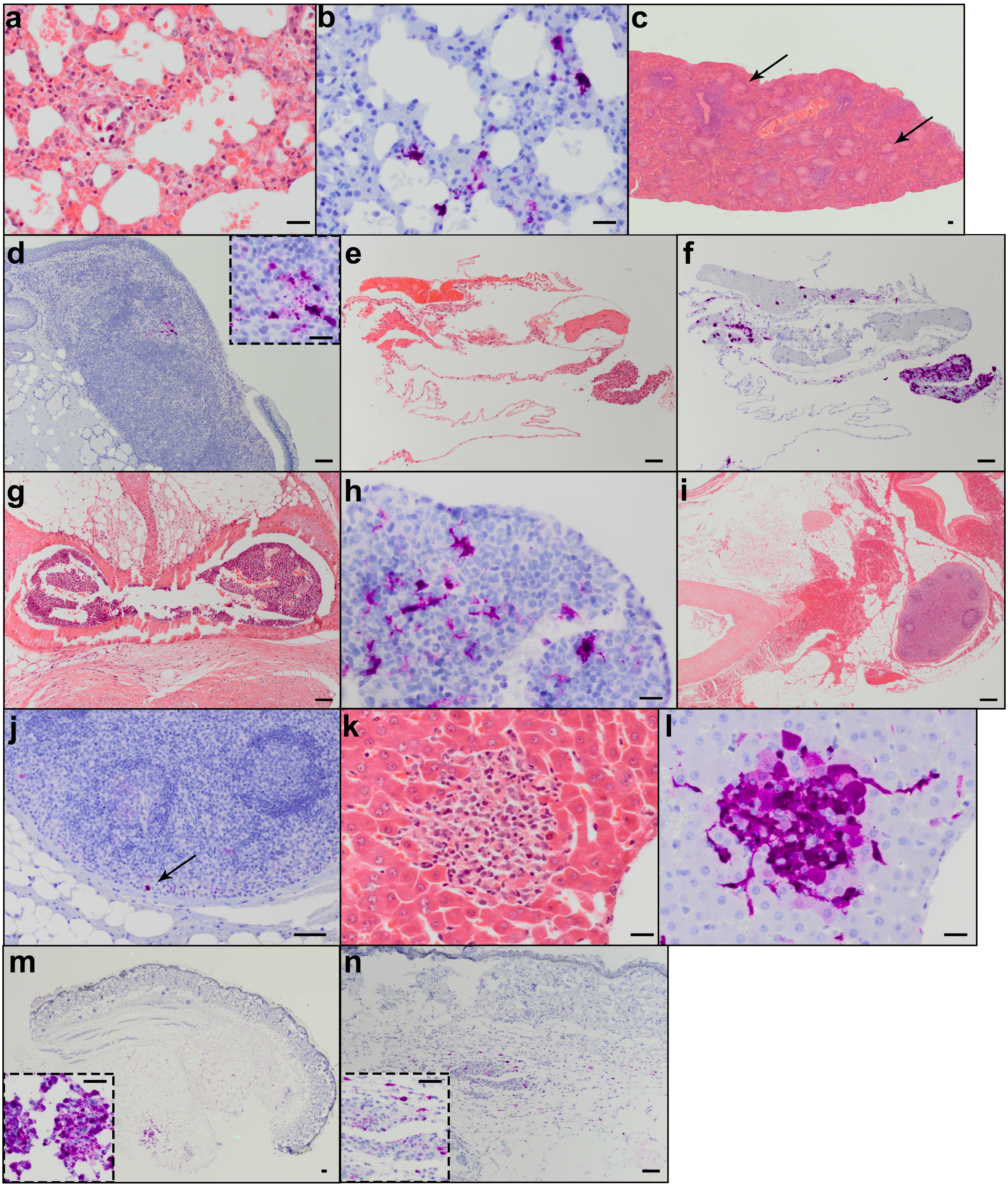

Supplementary Figure 6

Histology and filovirus-specific immunohistochemistry (IHC) images of experimentally filovirus-inoculated AFBs. a, Immune cell infiltration in lung tissue of bat E02, stained with haematoxylin and eosin (HE) **b,** and corresponding EBOV-antigen detection by IHC **c,** Normal spleen of a mock-inoculated control bat (HE); black arrows indicate islands of large histiocytic cells.

d, Tonsil tissue from bat E03 shows focally-restricted moderate EBOV immunostaining. **e**, Thymus fragment from EBOV-inoculated bat E03 (HE) and **f,** moderate IHC positive staining of numerous macrophages in the thymus and single scattered monocytes in (euthanasia-related) mediastinal haemorrhage. **g,** Bone marrow (hyoid bone) of bat E03 (HE) and **h**, multiple IHC positive cells in that tissue. **i,** Mesenteric lymph node of bat E02 with mild follicular hyperplasia (HE) and **j,** a single IHC positive macrophage (arrow). **k,** Liver of bat E03 showing focal necrosis (HE) **l,** Corresponding section of focal liver necrosis staining positive for EBOV-IHC **m,** IHC-positive cluster of macrophages in the subcutis at the skin inoculation site of EBOV-inoculated bat E01 and **n,** of RESTV-inoculated bat R02. **a-m,** micrographs are representative of one experiment with n=5 biologically independent animals. **n,** micrograph is representative of one experiment with n=6 biologically independent animals. Scale bars: 100 µm (c-g, i, j, m and n) and 25 µm (a, b, h, k, l and zoom-box).

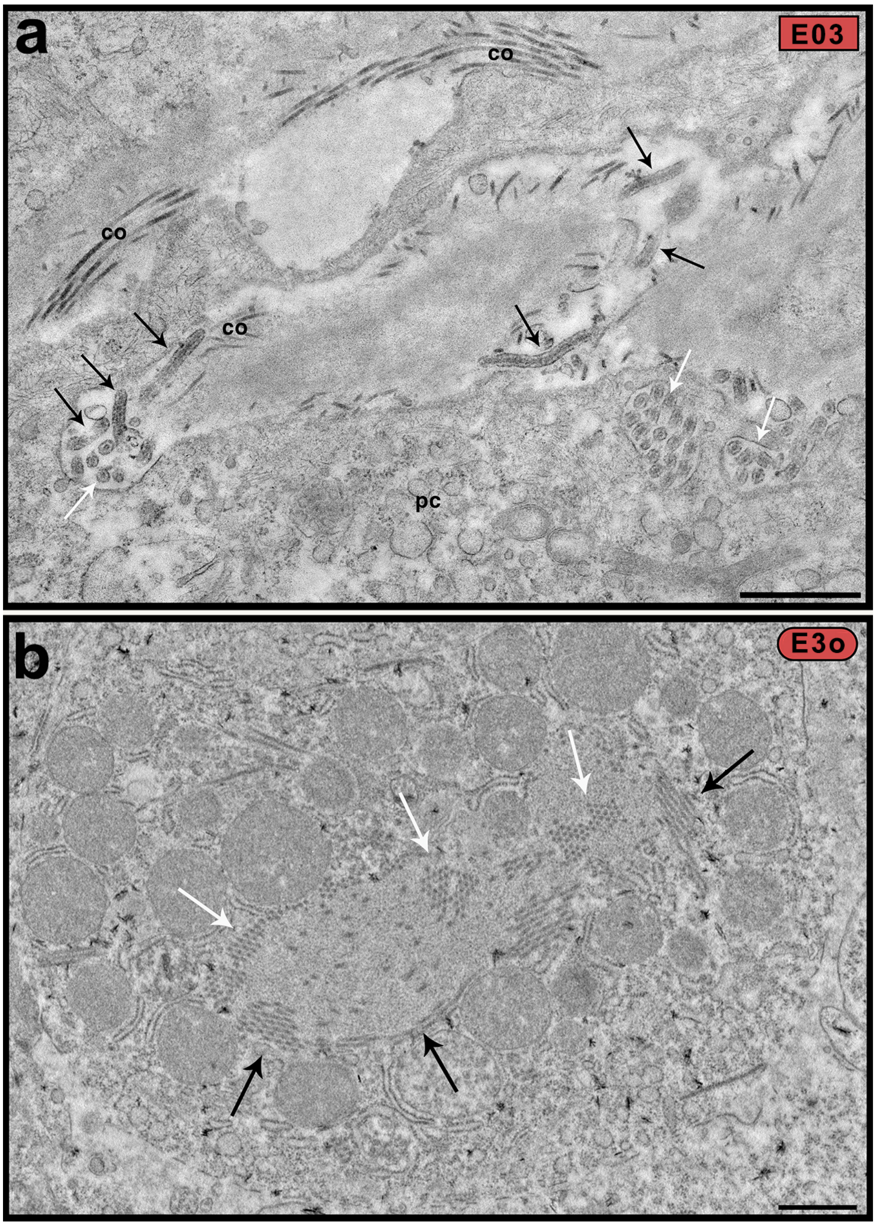

Supplementary Figure 7

Detection by thin section electron microscopy of EBOV virions and its replication in AFB placental and foetal tissues. a, Placenta of E03: EBOV particles are localized in the extracellular space, which is characterized by the presence of collagen (co), in close proximity to the placental cells (pc). Virus particles are shown in longitudinal (black arrows) or cross-section (white arrows) and reveal the typical nucleocapsid ribonucleoprotein (RNP) which appear as a hollow tube. **b,** E03's foetus, E3o: A foetal liver cell with a prominent cytoplasmic inclusion (virus factory) that reveals precursors of the nucleocapsid RNP in regular arrays embedded in a homogenous matrix (white arrows = cross-sections and black arrows = longitudinal or oblique sections through RNP). n= 4 biological independent animals from one experiment. Scale bar = 1 µm.

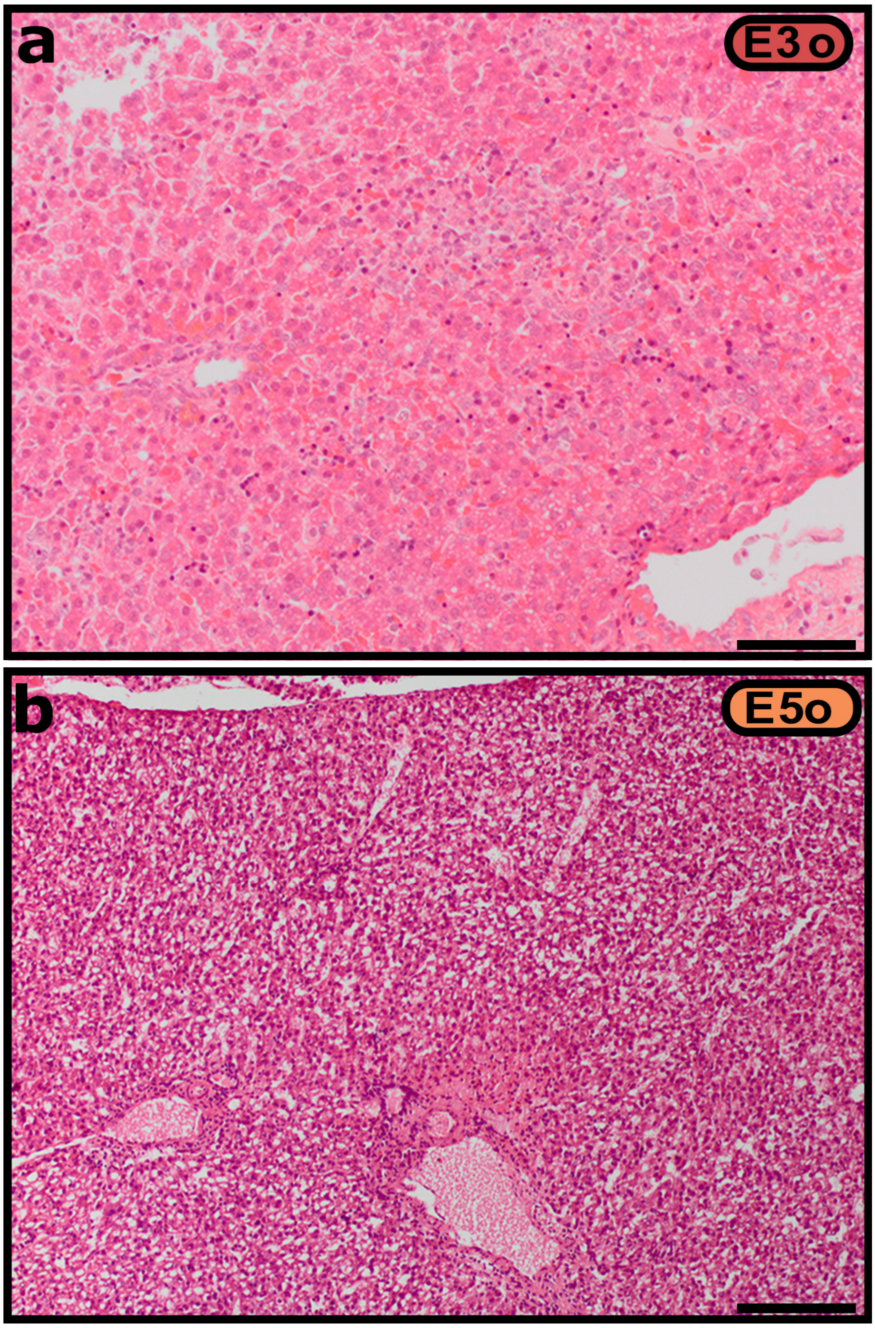

Supplementary Figure 8

Histopathology of foetus liver sections. a, E3o, foetus of dam E03. **b,** E5o, foetus of dam E05. n= 4 biological independent animals from one experiment. Scale bars: 50 µm

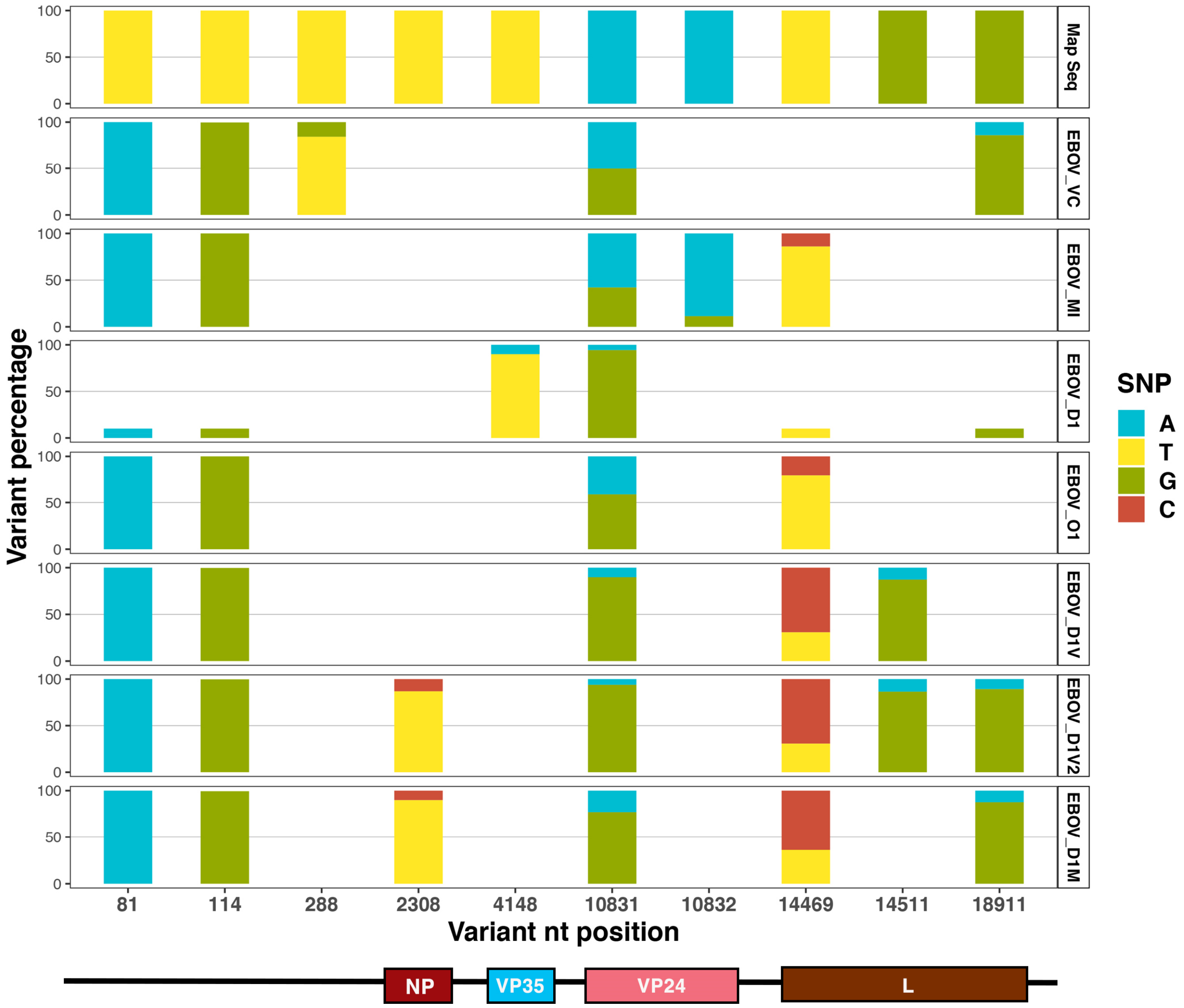

Supplementary Figure 9

Variant calling of sequence assemblies of EBOV isolates used in this study. Percentages of each variant, in reference to the mapping sequence, are shown in the y-axis and the positions of detected variants are shown in the x-axis. Below the x-axis, protein coding regions are depicted (coloured boxes) to match the variant site locations within EBOV genome. Map Seq= mapping sequence, EBOV_VC= original human isolate passaged in Vero cells, EBOV_MI= EBOV_VC passaged twice in MoKi cells and used as inoculum in the *in vivo* infection experiment, EBOV_D1= re-isolated virus obtained from kidney tissue of DE5, EBOV_O1= re-isolated virus from DE5's foetus, EBOV_D1V= EBOV_D1 passaged in Vero cells once, EBOV_D1V2= EBOV_D1 passaged in Vero cells twice, EBOV_D1M= EBOV_D1 passaged in MoKi cells once. No variant represents the same base as Map Seq and the 1% bar represents unknown base calling due to low coverage.

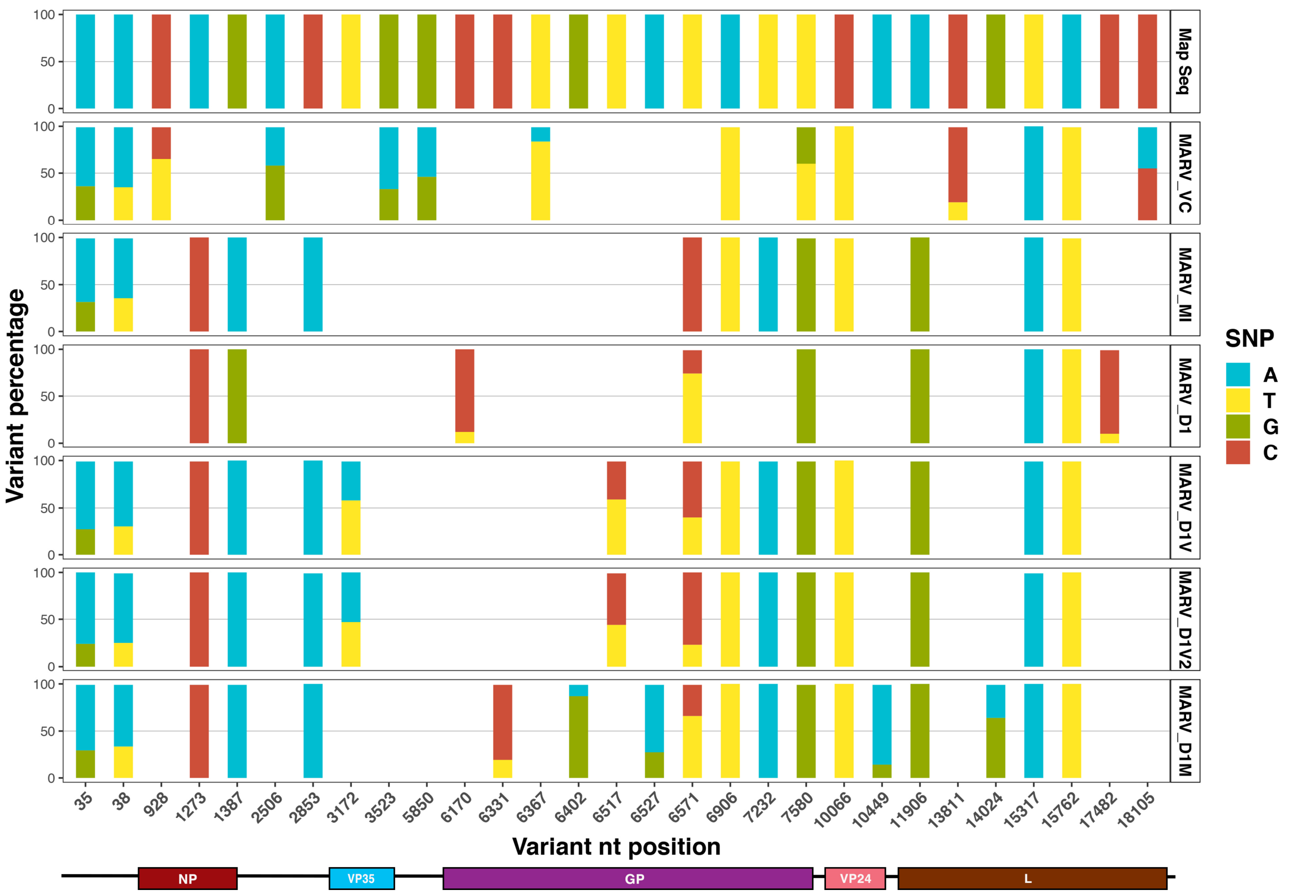

Supplementary Figure 10

Variant calling of sequence assemblies of MARV isolates used in this study. Percentages of each variant, in reference to the mapping sequence, are shown in the y-axis and the positions of detected variants are shown in the x-axis. Below the x-axis, protein coding regions are depicted (coloured boxes) to match the variant site locations within MARV genome. Map Seq= mapping sequence, MARV _VC= original human isolate passaged in Vero cells, MARV_MI= MARV_VC passaged twice in MoKi cells and used as inoculum in the *in vivo* infection experiment, MARV_D1= re-isolated virus obtained from placental tissue of M10, MARV_D1V= MARV_D1 passaged in Vero cells once, MARV_D1V2= MARV_D1 passaged in Vero cells twice, MARV_D1M= MARV_D1 passaged in MoKi cells once. No variant represents the same base as Map Seq.

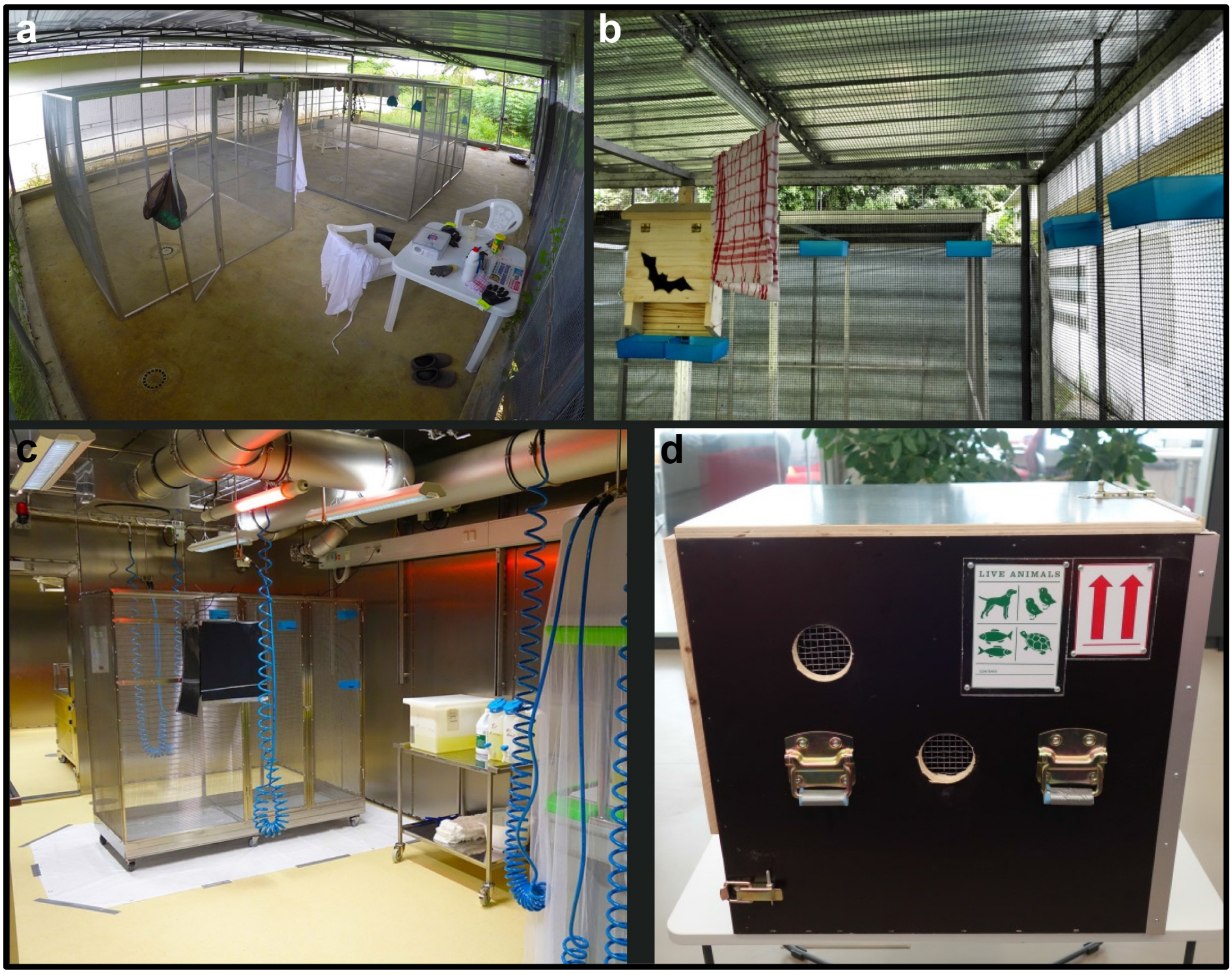

Supplementary Figure 11

Set up for AFBs captivity to experimental infection. a, Two flight cages were available for housing in captivity in Cote d'Ivoire. **b,** Bat houses, hanging cloths and hanging feeding dishes (blue) were included in the flight cages. **c,** Experimental enclosure system (including day and night - cages), which was set-up under biosafety level-4 conditions for the experimental infections. **d,** An IATA regulated outer transport box was constructed for the air transportation of the bats.

Supplementary Table 1

Details of the bat classification for the feeding pilot training.

Class	Description of activity during the pilot training
0	Bats feed on meal worms placed in the mouth individually.
1	Bats take the worms, self-feeding, from the forceps.
2	Worms are offered from different positions and are taken actively.
3	Worms are eaten directly by bat hanging at the cage grid.
4	Worms are eaten in a plastic box overnight; bats are self-feeding (bats that drop weight are supplemented with forceps feeding).
5	Worms are not handed to bats to train self-feeding from the dishes hanging at the cage grid (bats that drop weight are returned to previous step).
6	Bats eat autonomously from the dishes hanging at the cage grid

Supplementary Table 2

Details of the bat classification for the feeding optimized training.

Class	Description of activity during the optimization
0	Bats feed on meal worms placed in the mouth individually.
1	Bats take the worms, self-feeding, from the forceps.
2	Worms are offered from different positions and are taken actively.
3 + 4	Worms are fed (with forceps) directly from the dish, bat is inside the dish that is hanging from the cage grid
5	Worms are not handed to bats to train self-feeding from the dishes hanging at the cage grid.
6	Bats eat autonomously from the dishes hanging at the cage grid

Supplementary Table 3

Virus replication in Angolan free-tailed bats' kidney cell culture (MoKi). * TAFV inoculum was retrieved at MoKi-P3; **quantification of EBOV and MARV was done by 12 dpi

Virus	Inoculation volume for MoKi-P2	$TCID_{50}$/mL 11 dpi
EBOV C05 Makona	0.01µL	2.94×10^7 **
MARV Musoke	1µL	1.05×10^6 **
TAFV *	1µL	6.32×10^6
RESTV USA	1µL	1.50×10^7

Supplementary Table 4

Tissues collected and stored for downstream analyses. Samples for: q-RT-PCR stored in RLT buffer at -80 ºC, virus isolation samples stored natively at -80 ºC and histology stored in 10% formaldehyde at room temperature. ** Collection was done during necropsy and daily cage maintenance.

	Downstream Analyses		
Tissues	PCR	Cell Culture	Histology
Blood	✓	✓	×
Spleen	✓	✓	✓
Liver	✓	✓	✓
Gallbladder	✓	✓	✓
Stomach	✓	✓	✓
Small Intestine	✓	✓	✓
Colon	✓	✓	✓
Kidney	✓	✓	✓
Bladder	✓	✓	×
Reproductive Organs	✓	✓	×
Muscle	✓	✓	✓
Heart	✓	✓	✓
Lung	✓	✓	✓
B.A.T.	✓	✓	×
Skin Inoc.	✓	✓	✓
Bone Marrow	✓	×	×
Lymph Node	✓	✓	×
Eye	✓	✓	×
Brain	✓	✓	✓
Salivary Gland	✓	✓	×
Tongue	✓	✓	✓
**Maintenance buffer (PBS) ****			
Urine Swab		Day 2-10	
Feces		Day 2-10	
Oral Swab	✓	✓	
Rectal Swab	✓	✓	

Supplementary Table 5

AgPath-ID™ One-Step RT-PCR Kit assay used in this study

Reagent	Amount per reaction (µL)
Water	5,00
Buffer RT (2X)	12,50
Enhancer	1,00
F Primer 10 µM	1,00
R Primer 10 µM	1,00
Probe 10 µM	0,50
Enzyme Mix	1,00
Master Mix Volume	22,00
Sample Volume	3,00
Total Volume	25,00

Supplementary Table 6

RT-PCR primers and probes sequences and cycler conditions

Virus target	Sequence (5' – 3')	Cycler conditions
EBOV – VP30	FP: ACTCCTACTAATCGCCCGTAAG<br>RP: ATCAGCCGTTGGATTTGCT<br>PR: FAM-CACCCAAGGACTCGC-MGB	45°C x 900", 95°C x 600", (94°C x 15", 60°C x 45") x 45 cycles
MARV – L	FP: ACTCCTACTAATCGCCCGTAAG<br>RP: ATCAGCCGTTGGATTTGCT<br>PR: FAM-CCTATGCTTGCTGAATTGTGGTGCCA-BHQ1	45°C x 900", 95°C x 600", (94°C x 20", 59°C x 30") x 45 cycles
RESTV – L	FP: AAGGCCTTCCCCAGTAACATGAT<br>RP: GGCGGATTATAATAATCACTTACATGC<br>PR: FAM-CCAAAATCATCACTIGTGTGGTGCCA-BHQ1	
TAFV – L	FP: AAAGCATTCCCAAGCAATATGATGG<br>RP: GGGGGTTATAATAATCACTCACATG<br>PR: FAM-CCAAAATCATCACTIGTGTGGTGCCA-BHQ1	

Supplementary Table 7

Single nucleotide polymorphisms of MARV genome assembly detected against the original isolate passaged in Vero cells (MARV_VC), the mapping reference or in both. In bold are highlighted non-synonymous changes (dN). Minority variants (min-v) are described.

<table>
<tr><th>Region</th><th>Position</th><th>Compared to MARV_VC</th><th>Compared to mapping reference</th></tr>
<tr><td rowspan="3">NP CDS</td><td>928</td><td>dS change MARV_MI and subsequent isolates (T > C)</td><td>dS change in MARV_VC only (C > T), C is present as min-v</td></tr>
<tr><td>1273</td><td colspan="2">dN change in MARV_MI and subsequent isolates (A > C): E390D</td></tr>
<tr><td>1387</td><td colspan="2">dN change in MARV_MI and subsequent isolates (G > A): M428I</td></tr>
<tr><td rowspan="2">VP35 CDS</td><td>3172</td><td colspan="2">dS change in MARV_D1_V2 only, (T > A), T is present as min-v; A is also present as a min-v in MARV_D1_V</td></tr>
<tr><td>3523</td><td>dS change in MARV_MI and subsequent isolates (A > G)</td><td>dS change in MARV_VC only (G > A), G is present as min-v</td></tr>
<tr><td rowspan="5">GP CDS</td><td>6517</td><td colspan="2">dN change in MARV_D1_V2 only (T > C): S193P. T as min-v; C is also present as a min-v in MARV_D1_V</td></tr>
<tr><td>6571</td><td colspan="2">dN change in MARV_MI, MARV_D1V and MARV_D1V2 (T > C): C211R, with T as min-v in MARV_D1V and MARV_D1V2; C is also present as min-v in MARV_D1 and MARV_D1_M</td></tr>
<tr><td>6906</td><td></td><td>dS change in all isolates (A > T)</td></tr>
<tr><td>7232</td><td colspan="2">dN change in MARV_MI and subsequent isolates (T > A): F431Y</td></tr>
<tr><td>7580</td><td colspan="2">dN change in MARV_MI and subsequent isolates (T > G): V547G. G is also present as min-v in MARV_VC</td></tr>
<tr><td rowspan="3">L CDS</td><td>11906</td><td colspan="2">dS change in MARV_MI and subsequent isolates (A > G)</td></tr>
<tr><td>15317</td><td rowspan="2"></td><td>dS change in all isolates (G > A)</td></tr>
<tr><td>15762</td><td>dN change in all isolates (A > T): T1428S</td></tr>
<tr><td rowspan="5">non-coding</td><td>2506</td><td>MARV_MI and subsequent isolates (G > A)</td><td>MARV_VC only (A > G), A is present as min-v</td></tr>
<tr><td>2853</td><td colspan="2">MARV_MI and subsequent isolates (C > A)</td></tr>
<tr><td>5850</td><td>MARV_MI and subsequent isolates (A > G)</td><td>MARV_VC only (G > A), G as min-v</td></tr>
<tr><td>10066</td><td rowspan="2"></td><td>All isolates (C > T)</td></tr>
<tr><td>18682</td><td>All isolates (A insertion)</td></tr>
</table>

Supplementary Text

<u>Extended histopathology and immunohistochemistry findings not associated with infection</u>

Lympho-reticular tissue – HE staining:

In contrast to many other animals, AFBs possess distinct round to ovoid islands of large histiocytic cells surrounding the lymphoid follicles in a demarcating scaffold-like pattern (Fig. 3a, Fig. S6c arrows). In all animals, the lymphoid tissue of the spleen was unremarkable to mildly reactive with seven animals having mild follicular hyperplasia (Supplementary Data 3). Here, apoptotic cells were occasionally found in the reactive germinal centres of the follicles. Spleens of eight bats (EBOV, MARV and TAFV cohorts), were mildly infiltrated by neutrophils, accounting for systemic infection as leukostasis and increased numbers of progenitor cells of neutrophils were detected in other organs (i.e. lung and/or liver) and bone marrow (Fig. S6). In seven bats splenic extramedullary haematopoiesis occurred as it is commonly found in bats; three of these organs were noted as macroscopically enlarged or very enlarged. For 19 animals at least one or two lymph nodes were available (in descending order: retropharyngeal, mesenteric, pancreatic, mediastinal, renal, pulmonary), which either reflected the detected changes in the spleen of the respective animal or were without findings (Fig. S6c and i).

Lympho-reticular tissue – EBOV IHC staining:

The spleen of bat E03 contained numerous EBOV IHC-positive cells predominantly in the histiocytic cell islets surrounding lymphoid follicles, while the follicles themselves had only sparse EBOV-positive histiocytic cells (Fig. 3a). For the same bat, tonsils and thymus were available, one tonsil containing a small number of IHC-positive macrophages detected in the lymphoid center (Fig. S6d), while numerous EBOV-positive cells, most likely macrophages, were found in the thymus (Fig. S6e-f). In all three dpi-5 EBOV-inoculated bats (E01-E03) rare EBOV-positive monocytes were found intraluminal in larger blood vessels intermixed with erythrocytes and leukocytes of the peripheral blood. Similarly, sparse EBOV IHC-positive cells were detected in the bone marrow of bat E03 (Fig. S6g-h). For bats E02 and E03 mediastinal lymph nodes were sampled, each containing single distinctly EBOV-positive macrophages located in the subcapsular sinus (Fig. S6i-j).

Liver – HE staining:

Changes in the livers were of minor degree and in 14 animals restricted to lymphoplasmacytic infiltration of periportal spaces mixed with macrophages (n=10) and/or additional neutrophilic granulocytes (n=8), often alongside mild bile duct hyperplasia (n=7). Five bats (EBOV E01, E03,

MARV M01, M04, TAFV T01) had irregularly distributed degenerative to necrotic hepatocytes, three (EBOV E02, E03, MARV M01) distinct small foci of necrosis with small accumulations of macrophages, plasma cells and occasional lymphocytes (micro-abscesses) (Fig. 3a, Fig S6k), the other restricted to single cell necroses (MARV M04, TAFV T01). Except for three bats with unremarkable hepatocytes' cytoplasm all other animals' hepatocytes had fatty degeneration of varying degree (mild to severe) usually predominantly perilobular, only in severe cases expanding panlobularly.

Liver – EBOV IHC staining:

EBOV antigen-positive cells resembling Kupffer cells' morphology were found evenly disseminated throughout the liver in bats E02 and E03. Additionally, both bats had irregularly distributed single or small clusters of a few hepatocytes, which were positive for EBOV by IHC without evidence of marked immunoreaction. The small foci of necrosis in both bats noted in HE sections also proved to be EBOV-positive, specifically, degenerated hepatocytes and macrophages (Fig. 3a, Fig S6l). Additionally, scattered single EBOV IHC-positive hepatocytes were found. In contrast, observed periportal inflammatory infiltrations were EBOV-negative.

Lung – HE staining:

Blood vessels of almost all lungs were congested (Supplementary Data 3). The majority of bats from all inoculation groups and the control animals had mild pathological background changes such as infiltration of alveolar walls by neutrophilic granulocytes varying from sparse to moderate numbers (n=19), three of these animals also had evidence of leukostasis in their arterioles. Nine animals revealed mild to moderate pneumocyte hyperplasia of unknown cause. Two animals had mild hyperplasia of their pulmonary arterioles, suggestive of changes caused e.g. by microfilaria infection and one of the two control animals had mild suppurative bronchopneumonia.

Lung – EBOV IHC staining:

Only bat E02 had EBOV-positive cells in the lung (Fig. 3a). These were limited to a few lung lobuli containing multifocal macrophages in or next to alveolar walls and moderately to severely positive for EBOV (Fig. S6b).

Kidneys and abdominal cavity – HE staining:

Only two animals from the EBOV group had focal to multifocal mild interstitial infiltration by lymphocytes and plasma cells. One control animal had two renal tubules with intraluminal protozoal organisms. All other bats' kidneys were unremarkable. One EBOV-inoculated animal (E03) showed

focal infiltration by neutrophilic granulocytes of the renal adipose tissue adjacent to the renal pelvis. This animal also had further evidence of mild neutrophilic infiltration and multiple macrophages in the abdominal adipose tissue as well as focal accumulation of macrophages in adipose tissue at the splenic hilus, which was associated with positive IHC staining (Supplementary Data 3). Likewise, two bats of the TAFV group euthanized at 5 dpi had multifocal mild accumulations of macrophages, lymphocytes, plasma cells and neutrophils in the adipose tissue adjacent to spleen and jejunum.

Kidneys and abdominal cavity – EBOV IHC staining:

No EBOV-positive cells were detected in any of the kidneys (Supplementary Data 4). But EBOV-inoculated bat E03 had dense accumulations of distinctly EBOV IHC-positive macrophages in the abdominal adipose tissue adjacent to intestine, liver, kidney or spleen.

Skin – HE staining:

Skin sections comprising areas close to the inoculation site were available in 21 out of 25 animals (EBOV E01, E03, E05, MARV M01-M03, M05 and TAFV T01-T06, RESTV R01-R06 and mock controls C01-C02). Five of these bats (EBOV E01, TAFV T01, RESTV R01-R03) had evidence of small focal dermal/subcutaneous mild granulomatous inflammation.

Skin – EBOV IHC staining:

All subcutaneous inflammation sites of bats stained with IHC (EBOV E01, RESTV R01-R03) revealed antigen positive macrophages (Fig. S6m-n).

Gastrointestinal tract– HE staining:

The gastrointestinal tract was mostly unremarkable, except for animals with multifocal mild follicular lymphoplasmacytic accumulations in the gastric submucosa close to the lamina propria (n=8/18) and two animals with sparse infiltration of the lamina propria by additional neutrophilic granulocytes. Interestingly, five of the 18 bats consistently revealed coccoid bacteria restricted to the gastric pits close to the cardiac region of their stomachs. However, the number of animals with the same specific bacterial colonization might be greater as not all stomach tissue samples contained that region close to the oesophageal border. In two bats occasional nematode heads were burrowed in the superficial squamous epithelium of the oesophagus, in 11 bats the small intestine contained cross sections of different helminths (trematodes, nematodes, cestodes). When available, sections of large intestine (n=7) were unremarkable. Of the eighteen salivary glands available, two had mild interstitial lymphoplasmacytic infiltration, while the rest of the glands were without abnormal findings.

Gastrointestinal tract – EBOV IHC staining:
No positive EBOV IHC staining was detected in the gastrointestinal tract in any of the infection groups (5 dpi and 10 dpi).

Results genome assembly, EBOV:
Overall, the assemblies were very similar with only a small number of variants. After alignment of the consensus sequences, we detected four sites that had single nucleotide polymorphisms (SNPs) against the mapping reference (Fig. 5b). From which, only two were present against the original isolate passaged in Vero cells: EBOV_VC. All genomes differed from the reference in two sites (positions 81 T > A and 114 T > G, 100% frequency). Both sites are located in the non-coding region of the NP gene. The consensus themselves are almost identical but with differences in two positions against EBOV_VC (10831, 14469). For position 10831 (A > G), located in VP24 CDS, EBOV_MI and EBOV_VC are identical to the reference, while the other consensus genomes carry the variant A10831G, which leads to an amino acid (aa) substitution at position 163 of Lysine to Glutamic acid (K163E). However, it is notable that both variants (A and G) are present in all samples. While for EBOV_VC and EBOV_MI both variants are present to almost equal amounts (with reads containing A as a slight majority), the other samples (EBOV_D1, EBOV_D1M, EBOV_D1V, and EBOV_D1V2) have a clear majority of Gs in this site. In position 14469 (T > C) a synonymous change within L CDS, EBOV_VC, EBOV_MI, and EBOV_O1 match MG572232.1 (Map Seq), the other 4 samples do not (T14469C). Therefore, this transition was only reversed in the fetus of E03 (E3o) but not in other mammalian cell line passaging. In EBOV_VC this site is not variable. In all other samples both variants are present (although in EBOV_D1 the coverage is too low for variant calling).

Results genome assembly, MARV:
In comparison to EBOV, MARV sequence-assemblies varied up to 6.5 times more (SNPs detected against EBOV_VC and MARV_VC). After alignment of the consensus sequences we detected 18 sites with SNPs against the reference (Fig. 5d): 5 within non-coding regions and 13 within coding regions, from which 7 were non-synonymous (dN) and 6 were synonymous (dS). Against the original stock virus (MARV_VC), we detected 13 SNPs; 3 within non-coding regions and 10 within coding regions, from which 6 were dN and 4 were dS. Details of both comparisons are as shown in Table S7.
