# Supplementary material for: Selective replication and vertical transmission of Ebola virus in experimentally infected Angolan free-tailed bats
Source: Nat Commun. 2024 Jan 31;15:925. doi: 10.1038/s41467-024-45231-0 (PMC10830451; doi:10.1038/s41467-024-45231-0)
Supplement: Supplementary file 3 — Description of Additional Supplementary files [file 41467_2024_45231_MOESM3_ESM.pdf]

Description of additional Supplementary Information

File name: Supplementary Data 1
Description: Keys to identify Angolan free-tailed bats in Côte d'Ivoire and individual results of sequenced cyt b fragment of experimental bats

File name: Supplementary Data 2
Description: Macroscopic pathology findings score (0 to 2) during necropsy linked to IHC staining (score 0-3) in all cohorts

File name: Supplementary Data 3
Description: Individual animal parameters and histopathologic report for all cohorts linked to (partial) IHC results

File name: Supplementary Data 4
Description: Immunohistochemical findings
